# Prevalence, factors associated with use, and adverse effects of sport-related nutritional supplements (sport drinks, sport bars, sport gels): the US military dietary supplement use study

**DOI:** 10.1186/s12970-021-00457-x

**Published:** 2021-08-25

**Authors:** Joseph J. Knapik, Daniel W. Trone, Ryan A. Steelman, Emily K. Farina, Harris R. Lieberman

**Affiliations:** 1 Military Nutrition Division, US Army Research Institute of Environmental Medicine, 10 General Greene Ave, Natick, MA 01760 USA; 2 Henry Jackson Foundation for the Advancement of Military Medicine, Bethesda, MD USA; 3 Naval Health Research Center, San Diego, CA USA; 4 Army Public Health Center, Aberdeen Proving Ground, MD USA

**Keywords:** Demographics, Tobacco, Alcohol, Occupation

## Abstract

**Background:**

Sport-related nutritional supplements (SRNSs) include sport drinks, sport bars, and sport gels. Previous studies indicate that 25–35 % of athletes and 25–50 % of military personnel report using these supplements. This study examined prevalence, factors associated with use, and adverse effects (AEs) of SRNSs among United States military service members (SMs).

**Methods:**

A stratified random sample of 200,000 SMs was obtained from military workforce records, and asked to complete a survey on demographics, SRNS use, and AEs experienced. About 18 % (*n* = 26,681) of contacted SMs (*n* = 146,365) completed the survey between December 2018 and August 2019.

**Results:**

Overall, 45 % of SMs used ≥ 1 SRNS at least once per week in the past 6 months. Prevalence of use (± standard error) for sport drinks, bars, and gels were 32 ± 0.3, 27 ± 0.3, and 3 ± 0.1 %, respectively. Use of 1, 2, or 3 SRNSs was 28.9 ± 0.5, 13.6 ± 0.6, and 2.2 ± 0.6 %, respectively. Multivariable logistic regression indicated greater use of any SRNS was independently associated with male gender, younger age, single marital status, more weekly aerobic or resistance training, tobacco use, higher alcohol intake, officer status, combat arms occupations, and service in the Marine Corps or Navy (compared to the Air Force). Overall, the proportion of users reporting ≥ 1 AE was 2.0 ± 0.1 %, with 1.3 ± 0.1 % for sport drinks, 1.6 ± 0.2 % for sport bars, and 2.8 ± 0.6 % for sport gels.

**Conclusions:**

This large study of a stratified random sample of SMs found that nearly half of SMs consumed SRNSs weekly, and self-reported AEs were comparatively low. The AE incidence for SRNSs was much lower than typically found for dietary supplements, possibly because of more rigorous regulatory oversight for SRNSs.

## Background

Sport-related nutritional supplements (SRNSs) include sport drinks, sport bars, and sport gels. These substances are typically used before, during, or after exercise to provide hydration and/or nutrients. Sport drinks are generally carbohydrate-electrolyte solutions while sport bars and sport gels are generally composed of carbohydrate and protein complexes [1, 2]. SRNSs are commonly used by athletes and military personnel; about 25–35 % of athletes [3] and 25–50 % of military personnel [4–6] report using supplements of these types.

In the United States (US), regulation of SRNSs differs from that of dietary supplements because SRNSs are considered “food” and thus fall under the US Food and Drug Administration (FDA) regulations related to food [7]. In contrast, dietary supplements (DSs) fall under the Dietary Supplement Health and Education Act (DSHEA) of 1994, and the FDA has minimal power to regulate these substances [8]. Sport drink sales in 2018 amounted to $22.4 billion globally and $9.6 billion in the US [9, 10]. While some data suggest that sport drink use declined in the general US population from 2005 to 2011 [11], market projections suggest that US sales of sport drinks are expected to increase by 8 % over the period 2015–2025 [9, 10].

It is important to determine the prevalence of SRNS use to assess the wide-spread consumption of these substances. Further, examining demographic and lifestyle characteristics can provide information on who is using these supplements. Determining the incidence of adverse effects (AEs) associated with these supplements can assist in establishing the safety of these supplements. Previous studies have examined the prevalence of SRNS use in separate surveys of Army [4], Air Force [5], Navy and Marine Corps [6], and Coast Guard [12] personnel. The purpose of the current investigation was to perform a comprehensive examination of current SRNS use in a stratified random sample of all military services using the same survey instrument. This study expands on previous work by not only examining SRNS use prevalence, but also factors associated with use and self-reported AEs.

## Methods

This investigation involved a cross-sectional survey completed by US military service members (SMs) and was part of a larger study examining DS and SRNS use and AEs associated with their use [13]. The Naval Health Research Center’s institutional review board approved the investigation, and SMs consented to participate by signing an informed consent document. Investigators adhered to policies and procedures for protection of human subjects as prescribed by Department of Defense Instruction 3216.01, and the research was conducted in adherence with provisions of 32 Code of Federal Regulations Part 219.

### Sampling frame and solicitation procedures

Details of the sampling frame, solicitation of SMs, subject flow through the study, and response bias have been previously reported [13]. Briefly, investigators requested from the Defense Manpower Data Center (DMDC) a random sample of 200,000 SMs stratified by sex (88 % male and 12 % female) and branch of service (Army 36 %, Air Force 24 %, Marines 15 %, and Navy 25 %). Recruitment of SMs in this random sample involved a maximum of 12 sequential contacts. The prospective participant was first sent an introductory postal letter with a $1 pre-incentive designed to increase the response rate [14, 15]. The letter provided a link to a secure website where the SM could electronically sign the consent form and access the on-line survey. A follow-up email message after 10 days and a postcard after 3 weeks were sent as a reminder to those who did not initially complete the survey. If no response was received after sending the postcard, the subsequent contacts included up to seven email and three post card reminders evenly distributed over 8 months, after which contact with the SM ended. All postal and on-line reminder messages stated that at any time the SM could decline participation and be removed from the contact list. Recruitment began in December 2018, and data collection ended in August 2019.

### Survey description

The survey was based on previous questionnaires of this type [16] and designed to: (1) obtain type and frequency of SRNS use; (2) characterize participants so factors associated with use could be determined; and (3) ascertain AEs associated with use of SRNSs. To characterize participants, there were questions on demographics (gender, age, education, marital status, height, weight), lifestyle factors (amount of exercise, tobacco use, alcohol consumption), and military characteristics (rank, occupation assignment, service branch). There were four SRNS questions that asked SMs about the frequency of use and AEs associated with: (1) sport drinks; (2) sport bars; (3) sport gels; and (4) other. Commercial examples were provided for each SRNS (Table 1). The “other” entry was provided in case the participant could not categorize the SRNS and space was provided to enter the supplement name or type. For each SRNS, SMs were asked to estimate how frequently the supplement was consumed in the past 6 months (“never”, “once a month”, “once a week”, “2–6 times/week”, or “daily”). AEs on the survey were called “side effects,” and a list of AEs was located alongside each SRNS. The AE list included symptoms related to cardiovascular, gastrointestinal, muscular, sleep disturbance, and neurological symptoms. Specific symptoms listed on the survey included: “palpitations, racing heart”; “abdominal pain”; “nausea / vomiting”; “diarrhea”; “muscle cramps / pain / weakness”; “sleep disturbances / insomnia”; “dizzy / confusion / lightheaded”; “tingling / numb in extremities”; “seizures / convulsions / tremors”; and “other”. If “other” was selected, a space was provided for the SM to describe the experienced AE.

**Table 1:** Nutritional supplement type definitions

Type of Nutritional Supplement	Definition
Sport Drink	Liquids designed for use before, during, or after physical activity often containing carbohydrates and electrolytes. Examples provided on survey included Gatorade, Powerade, and G2.
Sport Bar	Solid substances designed to provide nutrients before, during, or after physical activity. Examples provided on survey included PowerBar, Clif Bar, and ProBar.
Sport Gel	Semi-solid substance designed to provide nutrients before, during or after activity. Examples provided on the survey included PowerBar Gel, GU, and Hammer Gel.
Sport Bar/Gel	Either sport bar or sport gel as defined above

### Data analysis

All statistical analyses were conducted using the Statistical Package for the Social Sciences (Version 26, 2019, SPSS Inc.). Body mass index (BMI) was computed from the survey responses as weight/height^2^ (kg/m^2^). Weekly duration of aerobic and resistance training (min/week) was calculated by multiplying reported weekly exercise frequency (sessions/week) by the reported duration of training (min/session). Alcohol consumption was quantified under the National Institute of Health assumption that a “standard drink” contained 17.74 ml of alcohol [17]. “Standard drinks” included 12 oz of regular beer or fermented fruit drink (5 % alcohol), 8.5 oz of higher alcohol beer (7 % alcohol), 5 oz of wine (12 % alcohol), 4.25 oz of fortified wine (15 % alcohol), and 1.5 oz of liquor (40 % alcohol). If a SM listed “other” for a SRNS, these were individually examined and placed into the proper category (Table 1).

Prevalence (as a %) and standard error (SE) were calculated for each SRNS and AE (i.e., %±SE). Chi-square statistics examined differences in prevalence across various strata of demographic, lifestyle, and military characteristics as well as the number of SRNSs used. Where variables were ordinal (i.e., age, education, BMI, aerobic training duration, resistance training duration, and alcohol intake), chi-square tests for linear trend (Mantel-Haenszel statistic) were also performed. Multivariable logistic regression examined associations between the dependent SRNS use (yes/no) and independent variables involving demographic, lifestyle, and military characteristics. Odds ratios (OR) and 95 % confidence intervals (95 %CI) were calculated comparing a reference stratum (defined with an OR = 1.00) to other strata within that variable. Since logistic regression requires full data on all variables, only participants who completed all demographic, lifestyle, and military characteristics could be included in these analyses. Some participants did not complete all questions, so the number of subjects are shown for each variable in tables.

## Results

From the initial sample frame of 200,000 SMs, 73 % (*n* = 146,365) were successfully contacted (i.e., no returned postal mail) and of these, 26,681 (18.2 %) signed the informed consent and completed the survey.

### Overall prevalence

Table 2 provides the overall prevalence and number of SRNSs consumed by SMs during the past 6 months. Forty-five percent (45 %) of SMs reported using ≥ 1 SRNS at least one time per week. Sport drinks, sport bars, and sport gels were used by 32, 27 and 3 % of SMs, respectively; most users consumed only 1 of the 3 types of SRNSs. The last column in Table 2 represents SMs who consumed either sport bars or sport gels (sport bars/gels). This combined use type was computed to compare with other studies which combine these categories [4–6, 12].

**Table 2:** Prevalence of sport-related nutritional supplement use reported by service members by demographic, lifestyle, and military characteristics

Variable	Strata	Any SRNS Consumed ≥1 Time per Week (%±SE)	Number of SRNSs Consumed ≥1 Time per Week (%±SE)			Specific SRNS Types Consumed ≥1 Time per Week (%±SE)			
			1	2	3	Sport Drink	Sport Bar	Sport Gel	Sport Bar & Gel^a^
Cohort	All (*n* = 26,681)	44.6 ± 0.5	28.9 ± 0.5	13.6 ± 0.6	2.2 ± 0.6	32.3 ± 0.3	26.9 ± 0.3	3.4 ± 0.1	27.5 ± 0.3
Gender	Men (*n* = 23,038)	46.1 ± 0.3	29.6 ± 0.6	14.3 ± 0.6	2.3 ± 0.7	33.7 ± 0.3	27.7 ± 0.3	3.5 ± 0.1	28.4 ± 0.3
	Women (*n* = 3,642)	35.0 ± 0.8	24.2 ± 1.4	9.3 ± 1.6	1.4 ± 1.6	23.1 ± 0.7	21.3 ± 0.7	2.7 ± 0.3	21.9 ± 0.7
	*p*-value (chi-square)	<0.01	<0.01			<0.01	<0.01	0.01	<0.01
Age	18-24 yr (*n* = 4,660)	50.8 ± 0.7	30.6 ± 1.2	17.9 ± 1.3	2.3 ± 1.5	41.8 ± 0.7	28.6 ± 0.7	2.8 ± 0.2	29.9 ± 0.7
	25-29 yr (*n* = 5,580)	46.2 ± 0.7	29.3 ± 1.1	14.7 ± 1.2	2.2 ± 1.3	33.5 ± 0.6	28.6 ± 0.6	3.2 ± 0.2	29.1 ± 0.6
	30-39 yr (*n* = 11,031)	43.7 ± 0.5	29.0 ± 0.8	12.6 ± 0.9	2.1 ± 0.9	30.3 ± 0.4	26.8 ± 0.4	3.5 ± 0.2	27.6 ± 0.4
	≥40 yr (*n* = 5,275)	39.8 ± 0.7	26.9 ± 1.2	10.7 ± 1.3	2.1 ± 1.4	27.1 ± 0.6	23.9 ± 0.6	3.7 ± 0.6	24.9 ± 0.6
	*p*-value (chi square/trend)	<0.01/<0.01	<0.01/<0.01			<0.01/<0.01	<0.01/<0.01	0.06/<0.01	<0.01/<0.01
Formal Education	Some HS/HS Grad (*n* = 3,879)	46.9 ± 0.8	29.5 ± 1.3	15.1 ± 1.5	2.3 ± 1.6	39.9 ± 0.8	24.0 ± 0.7	2.9 ± 0.3	24.4 ± 0.7
	Some College (*n* = 11,378)	44.3 ± 0.5	28.8 ± 0.8	13.8 ± 0.9	1.8 ± 0.9	34.2 ± 0.4	24.7 ± 0.4	2.8 ± 0.2	25.2 ± 0.4
	College Degree (*n* = 11,418)	44.1 ± 0.5	28.8 ± 0.8	12.9 ± 0.9	2.4 ± 0.9	27.8 ± 0.4	30.0 ± 0.4	4.0 ± 0.2	30.8 ± 0.4
	*p*-value (chi square/trend)	<0.01/<0.01	<0.01/<0.01			<0.01/<0.01	<0.01/<0.01	<0.01/<0.01	<0.01/<0.01
Marital Status	Never Married (*n* = 6,471)	43.2 ± 0.4	28.4 ± 0.6	12.6 ± 0.7	2.1 ± 0.7	37.3 ± 0.6	26.1 ± 0.5	3.5 ± 0.2	26.9 ± 0.6
	Married (*n* = 17,686)	48.8 ± 0.6	30.2 ± 1.0	16.4 ± 1.1	2.2 ± 1.2	30.5 ± 0.3	29.3 ± 0.3	3.0 ± 0.1	29.6 ± 0.6
	Sep, Widowed, Divorced (*n* = 2,145)	43.5 ± 1.1	28.8 ± 1.8	12.8 ± 2.0	2.0 ± 2.1	31.2 ± 1.0	26.0 ± 0.9	3.1 ± 0.4	26.6 ± 1.0
	*p*-value (chi-square)	<0.01	<0.01			<0.01	<0.01	0.17	<0.01
Body Mass Index	<25.0 kg/m^2^ (*n* = 7,856)	44.0 ± 0.6	28.5 ± 1.0	13.2 ± 1.1	2.3 ± 1.1	31.8 ± 0.5	26.3 ± 0.5	3.6 ± 0.2	26.9 ± 0.5
	25.0-29.9 kg/m^2^ (*n* = 13,898)	45.2 ± 0.4	29.1 ± 0.7	14.0 ± 0.8	2.1 ± 0.8	32.2 ± 0.4	28.0 ± 0.4	3.3 ± 0.2	28.7 ± 0.4
	≥30.0 kg/m^2^ (*n* = 4,424)	44.3 ± 0.7	29.3 ± 1.3	13.0 ± 1.4	2.0 ± 1.5	33.5 ± 0.7	24.7 ± 0.6	3.2 ± 0.3	25.3 ± 0.7
	*p*-value (chi square/trend)	0.17/0.50	0.26/0.95			0.18/0.09	<0.01/0.26	0.37/0.16	<0.01/0.31
Aerobic Exercise Duration	≤90 min/wk (*n* = 7,287)	38.9 ± 0.6	27.2 ± 1.0	10.7 ± 1.1	1.0 ± 1.2	27.1 ± 0.5	23.2 ± 0.5	1.4 ± 0.1	23.4 ± 0.5
	91-180 min/wk (*n* = 7,285)	43.9 ± 0.6	29.4 ± 1.0	13.0 ± 1.1	1.6 ± 1.2	31.6 ± 0.5	26.3 ± 0.5	2.2 ± 0.2	26.6 ± 0.5
	181-300 min/wk (*n* = 5,869)	46.7 ± 0.7	29.9 ± 1.1	14.6 ± 1.2	2.2 ± 1.3	33.3 ± 0.6	28.5 ± 0.6	3.9 ± 0.3	29.3 ± 0.6
	>300 min/wk (*n* = 6,240)	50.0 ± 0.6	29.3 ± 1.1	16.6 ± 1.2	4.2 ± 1.2	38.2 ± 0.6	30.3 ± 0.6	6.5 ± 0.3	31.7 ± 0.6
	*p*-value (chi square/trend)	<0.01/<0.01	<0.01/<0.01			<0.01/<0.01	<0.01/<0.01	<0.01/<0.01	<0.01/<0.01
Resistance Training Duration	≤45 min/wk (*n* = 7,776)	34.9 ± 0.5	23.9 ± 1.0	9.6 ± 1.1	1.4 ± 1.1	26.8 ± 0.5	18.0 ± 0.4	2.6 ± 0.2	18.7 ± 0.4
	46-135 min/wk (*n* = 6,257)	43.9 ± 0.6	29.8 ± 1.1	12.0 ± 1.2	2.0 ± 1.2	31.4 ± 0.6	25.3 ± 0.6	3.3 ± 0.2	26.0 ± 0.6
	136-300 min/wk (*n* = 6,582)	49.8 ± 0.6	31.9 ± 1.0	15.8 ± 1.1	2.1 ± 1.2	34.9 ± 0.6	31.7 ± 0.6	3.3 ± 0.2	32.3 ± 0.6
	>300 min/wk (*n* = 6,066)	52.1 ± 0.6	31.0 ± 1.1	17.9 ± 1.2	3.2 ± 1.3	37.1 ± 0.6	34.6 ± 0.6	4.5 ± 0.3	35.2 ± 0.6
	*p*-value (chi-square/trend)	<0.01/<0.01	<0.01/<0.01			<0.01/<0.01	<0.01/<0.01	<0.01/<0.01	<0.01/<0.01
Smoking	Never Smoked (*n* = 16,706)	42.7 ± 0.4	27.9 ± 0.7	12.6 ± 0.7	2.2 ± 0.8	30.0 ± 0.4	26.3 ± 0.3	3.3 ± 0.1	26.9 ± 0.3
	Smoked but Quit (*N* = 4,767)	46.1 ± 0.7	29.7 ± 1.2	14.4 ± 1.3	2.0 ± 1.5	33.1 ± 0.7	27.6 ± 0.6	3.6 ± 0.3	28.6 ± 0.7
	Smoker (*n* = 4,512)	51.4 ± 0.7	32.4 ± 1.2	16.8 ± 1.4	2.3 ± 1.5	40.4 ± 0.7	29.1 ± 0.7	3.3 ± 0.3	29.7 ± 0.7
	*p*-value (chi-square)	<0.01	<0.01			<0.01	<0.01	0.70	<0.01
Smokeless Tobacco Use	Never Used (*n* = 20,379)	42.6 ± 0.3	28.2 ± 0.6	12.4 ± 0.7	2.0 ± 0.7	30.4 ± 0.3	25.5 ± 0.3	3.1 ± 0.1	26.1 ± 0.3
	Used but Quit (*n* = 2,047)	51.9 ± 1.1	31.7 ± 1.8	17.4 ± 2.0	2.8 ± 2.2	36.5 ± 1.1	33.7 ± 1.1	4.7 ± 0.5	34.7 ± 1.1
	User (*n* = 3,114)	55.6 ± 0.9	33.0 ± 1.5	19.7 ± 1.6	2.9 ± 1.8	43.6 ± 09	33.4 ± 0.8	4.0 ± 0.4	34.1 ± 0.8
	*p*-value (chi-square)	<0.01	<0.01			<0.01	<0.01	<0.01	<0.01
Alcohol Intake	0 ml/wk (*n* = 8,372)	37.3 ± 0.5	24.8 ± 0.9	10.7 ± 1.0	1.7 ± 1.1	27.9 ± 0.5	21.0 ± 0.4	2.7 ± 0.2	21.5 ± 0.4
	0.23-24.85 ml/wk (*n* = 6,132)	41.3 ± 0.6	28.4 ± 1.1	11.3 ± 1.2	1.7 ± 1.3	28.5 ± 0.6	24.5 ± 0.5	2.9 ± 0.2	25.1 ± 0.6
	24.86-71.69 ml/wk (*n* = 6,108)	49.5 ± 0.6	31.9 ± 1.1	15.4 ± 1.2	2.2 ± 1.3	35.0 ± 0.6	30.7 ± 0.6	3.6 ± 0.2	31.6 ± 0.6
	≥71.70 ml/wk (*n* = 6,068)	53.0 ± 0.6	32.0 ± 1.1	18.0 ± 1.2	3.1 ± 1.3	39.3 ± 0.6	33.4 ± 0.6	4.5 ± 0.3	34.2 ± 0.6
	*p*-value (chi square/trend)	<0.01/<0.01	<0.01/<0.01			<0.01/<0.01	<0.01/<0.01	<0.01/<0.01	<0.01/<0.01
Rank	Junior Enlisted (*n* = 2,496)	47.4 ± 1.0	29.4 ± 1.7	15.8 ± 1.8	2.1 ± 2.0	39.1 ± 1.0	26.0 ± 0.9	2.4 ± 0.3	26.1 ± 0.9
	Mid-Enlisted (*n* = 11,609)	44.3 ± 0.5	28.9 ± 0.8	13.5 ± 0.9	1.8 ± 0.9	34.5 ± 0.4	24.2 ± 0.4	2.7 ± 0.2	24.7 ± 0.4
	Senior Enlisted (*n* = 4,365)	41.5 ± 0.7	26.8 ± 1.3	12.3 ± 1.4	2.3 ± 1.5	30.9 ± 0.4	23.8 ± 0.4	3.8 ± 0.2	24.6 ± 0.7
	Warrant Officer (*n* = 576)	43.4 ± 2.1	29.3 ± 3.5	11.5 ± 3.9	2.6 ± 4.1	29.2 ± 1.8	26.0 ± 1.8	4.9 ± 0.9	27.3 ± 1.8
	Junior Officer (*n* = 3,892)	49.3 ± 0.8	30.8 ± 1.3	15.7 ± 1.5	2.8 ± 1.6	30.7 ± 0.7	35.8 ± 0.8	4.2 ± 0.3	36.3 ± 0.8
	Senior Officer (*n* = 3,742)	42.7 ± 0.8	28.6 ± 1.4	11.9 ± 1.5	2.2 ± 1.6	24.5 ± 0.7	30.1 ± 0.8	4.4 ± 0.3	31.4 ± 0.8
	*p*-value (chi-square)	<0.01	<0.01			<0.01	<0.01	<0.01	<0.01
Occupational Assignment Group	Combat Arms (*n* = 6,451)	50.3 ± 0.6	30.6 ± 1.0	16.5 ± 1.1	3.2 ± 1.2	36.1 ± 0.6	32.5 ± 0.6	4.7 ± 0.3	33.3 ± 0.6
	Combat Support (*n* = 10,424)	43.9 ± 0.5	28.5 ± 0.8	13.5 ± 0.9	2.0 ± 1.0	31.9 ± 0.5	26.3 ± 0.4	3.1 ± 0.2	26.8 ± 0.4
	Combat Service Support (*n* = 9,133)	41.9 ± 0.5	28.3 ± 0.9	11.9 ± 1.0	1.7 ± 1.0	30.4 ± 0.5	24.1 ± 0.4	2.8 ± 0.2	24.7 ± 0.5
	*p*-value (chi-square)	<0.01	<0.01			<0.01	<0.01	<0.01	<0.01
Service Branch	Air Force (*n* = 9,789)	41.3 ± 0.5	28.2 ± 0.9	11.4 ± 1.0	1.7 ± 1.0	29.0 ± 0.5	24.5 ± 0.4	2.6 ± 0.2	25.0 ± 0.4
	Army (*n* = 7,935)	45.1 ± 0.6	29.3 ± 0.9	13.6 ± 1.1	2.3 ± 1.1	33.6 ± 0.5	25.6 ± 0.5	3.8 ± 0.2	26.8 ± 0.5
	Marine Corps (*n* = 3,194)	54.3 ± 0.9	31.1 ± 1.5	19.8 ± 1.6	3.5 ± 1.7	42.5 ± 0.9	34.0 ± 0.8	4.5 ± 0.4	34.6 ± 0.8
	Navy (*n* = 5,763)	44.0 ± 0.7	28.1 ± 1.1	13.8 ± 1.2	2.1 ± 2.0	30.2 ± 0.6	28.3 ± 0.6	3.5 ± 0.2	28.9 ± 0.6
	*p*-value (chi-square)	<0.01	<0.01			<0.01	<0.01	<0.01	<0.01

Sums may not equal the total sample size due to missing data (SMs did not answer all questions)

*Abbreviations*: *SRNS* sports-related nutritional supplement, *SE* Standard error, *HS* High School, *Grad* Graduate, *Sep* Separated

^a^Service member used either sports bar or sports gel. Included because many military studies combine these supplement types [4–6, 12]

### Factors associated with SRNS use

Table 2 also presents demographic, lifestyle, and military factors related to SRNS use. Compared to women, men reported using a greater number of SRNS and had higher use among all three types. As age increased, the number of SRNSs used and the use of sport drinks and sport bars decreased, while sport gel use demonstrated a linear trend increasing with age. The number of SRNSs used and sport drink use decreased as education increased, while use of sport bars and gels generally increased as education increased. SMs who had never married were more likely to use sport drinks, while married SMs used a greater number of SRNSs and used more sport bars. BMI had little association with the number of SRNSs used or use of sport drinks or sport gels, but sport bar use was highest among those with a BMI of 25.0-29.9 kg/m^2^. As the amount of aerobic or resistance training increased, so did the number of SRNSs used and use of all 3 types of SRNSs. Smokers were more likely to use a greater number of SRNSs as well as have higher use of sport drinks and bars. Smokeless tobacco users used the largest number of SRNSs and had higher use of sport drinks, while those who were former smokeless tobacco users had the highest use of sport bars and gels. As alcohol consumption increased, so did the number and use of all three types of SRNSs. Enlisted personnel were more likely to use sport drinks, but officers were more likely to use sport bars and gels; junior officers used the greatest number of SRNSs. SMs in combat arms occupations used the greatest number of SRNSs and had the highest use of all three SRNS types. Marine Corps personnel used the greatest number of SRNSs and had the highest use prevalence of all three types of SRNSs. Air Force personnel used the lowest number of SRNSs and had the lowest use of all three SRNS types.

Table 3 presents results of the multivariable logistic regression examining factors associated with SRNS use. There were 24,010 SMs with complete data on all variables, so 90.0 % of SMs were included in this analysis. Factors independently associated with use of any SRNS included male gender, younger age, being single, performing more aerobic or resistance training, being a smoker, being a current or former smokeless tobacco user, higher alcohol intake, officer status, being in combat arms occupations, and service in the Marine Corps or Navy (compared to the Air Force). Sport drink use was independently associated with male gender, younger age, less education, being single, more aerobic or resistance training, being a smoker, being a former or current smokeless tobacco user, higher alcohol intake, junior enlisted status (compared to senior officer status), combat arms occupations, and service in the Army, Marine Corps, or Navy (compared to the Air Force). Factors independently associated with use of sport bars included male gender, younger age, higher education, more aerobic or resistance training, being a current or former smokeless tobacco user, higher alcohol intake, officer status, combat arms occupations, and service in the Marine Corps or Navy (compared to the Air Force). Sport gel use was independently associated with more aerobic exercise, being a former smokeless tobacco user, higher alcohol intake, warrant officer or senior officer status (compared to junior enlisted), combat arms occupations, and service in the Marine Corps or Navy (compared to the Air Force). Results for SMs who consumed combined sport bars or gels were similar to sport bars alone, as would be expected because of the larger prevalence of sport bar use.

**Table 3:** Multivariable analysis (logistic regression) of factors associated with sport-related nutritional supplement use by service members

Variable	Strata	Odds Ratio (95% Confidence Interval)				
		Any SRNS	Sport Drink	Sport Bar	Sport Gel	Sport Bar & Gel^a^
Gender	Male (*n* = 20,728)	1.00	1.00	1.00	1.00	1.00
	Female (3,282)	0.80 (0.73–0.87)	0.72 (0.66–0.79)	0.91 (0.82–0.99)	0.95 (0.75–1.20)	0.92 (0.86–0.98)
Age	18–24 yr (*n* = 4,165)	1.00	1.00	1.00	1.00	1.00
	25–29 yr (*n* = 5,091)	0.76 (0.69–0.85)	0.73 (0.66–0.80)	0.83 (0.74–0.92)	0.97 (0.74–1.28)	0.82 (0.74–0.92)
	30–39 yr (*n* = 10,003)	0.71 (0.64–0.79)	0.67 (0.61–0.75)	0.75 (0.67–0.84)	0.95 (0.71–1.27)	0.76 (0.67–0.85)
	≥40 yr (*n* = 4,751)	0.61 (0.54–0.70)	0.64 (0.56–0.73)	0.62 (0.53–0.71)	0.88 (0.62–1.24)	0.62 (0.54–0.71)
Formal Education	Some HS/HS Grad (*n* = 3,381)	1.00	1.00	1.00	1.00	1.00
	Some College (*n* = 10,257)	1.09 (0.99–1.20)	0.99 (0.90–1.08)	1.25 (1.13–1.38)	1.01 (0.78–1.30)	1.24 (1.12–1.38)
	College Degree (*n* = 10,372)	1.09 (0.97–1.22)	0.87 (0.77–0.98)	1.45 (1.28–1.65)	1.21 (0.89–1.65)	1.45 (1.27–1.65)
Marital Status	Married (*n* = 16,143)	1.00	1.00	1.00	1.00	1.00
	Never Married (*n* = 5,922)	1.11 (1.03–1.19)	1.12 (1.04–1.21)	1.08 (0.99–1.17)	0.96 (0.78–1.17)	1.07 (0.99–1.16)
	Sep, Widowed, Divorced (*n* = 1,945)	1.02 (0.92–1.13)	1.04 (0.94–1.16)	1.00 (0.90–1.12)	0.91 (0.69–1.20)	1.00 (0.89–1.11)
Body Mass Index	<25.0 kg/m^2^(*n* = 7,279)	1.00	1.00	1.00	1.00	1.00
	25.0–29.9 kg/m^2^ (*n* = 12,722)	1.00 (0.94–1.07)	1.00 (0.93–1.06)	1.02 (0.95–1.09)	0.85 (0.73–1.01)	1.02 (0.96–1.10)
	≥30.0 kg/m^2^(*n* = 4,009)	1.02 (0.94–1.11)	1.08 (0.99–1.17)	0.93 (0.84–1.02)	0.82 (0.65–1.03)	0.93 (0.85–1.03)
Aerobic Exercise Duration	≤90 min/wk (*n* = 6,240)	1.00	1.00	1.00	1.00	1.00
	91–180 min/wk (*n* = 6,656)	1.21 (1.13–1.30)	1.29 (1.19–1.39)	1.15 (1.06–1.25)	1.81 (1.37–2.38)	1.15 (1.06–1.25)
	181–300 min/wk (*n* = 5,387)	1.26 (1.17–1.36)	1.32 (1.21–1.44)	1.19 (1.09–1.29)	3.13 (2.40–4.08)	1.23 (1.12–1.34)
	>300 min/wk (*n* = 5,727)	1.34 (1.12–1.45)	1.56 (1.43–1.70)	1.16 (1.06–1.27)	5.82 (4.50–7.53)	1.25 (1.14–1.36)
Resistance Training Duration	≤45 min/wk (*n* = 6,575)	1.00	1.00	1.00	1.00	1.00
	46–135 min/wk (*n* = 5,735)	1.38 (1.28–1.48)	1.15 (1.06–1.24)	1.50 (1.38–1.64)	1.11 (0.89–1.37)	1.48 (1.36–1.62)
	136–300 min/wk (*n* = 6,063)	1.64 (1.52–1.77)	1.21 (1.12–1.31)	2.04 (1.87–2.22)	0.95 (0.77–1.18)	1.98 (1.81–2.16)
	>300 min/wk (*n* = 5,623)	1.72 (1.58–1.87)	1.13 (1.04–1.23)	2.53 (2.30–2.79)	1.07 (0.85–1.34)	2.42 (2.21–2.66)
Smoking	Never Smoked (*n* = 15,511)	1.00	1.00	1.00	1.00	1.00
	Smoked but Quit (*n* = 4,337)	1.05 (0.97–1.13)	1.01 (0.94–1.10)	1.04 (0.96–1.14)	0.97 (0.79–1.19)	1.06 (0.97–1.15)
	Smoker (*n* = 4,162)	1.14 (1.06–1.23)	1.20 (1.11–1.29)	0.98 (0.91–1.07)	0.86 (0.70–1.06)	0.99 (0.91–1.07)
Smokeless Tobacco Use	Never Used (*n* = 19,143)	1.00	1.00	1.00	1.00	1.00
	Used but Quit (*n* = 1,924)	1.23 (1.11–1.36)	1.11 (1.00–1.23)	1.34 (1.20–1.49)	1.33 (1.04–1.70)	1.35 (1.21–1.51)
	User (2,943)	1.32 (1.21–1.43)	1.33 (1.21–1.44)	1.27 (1.16–1.39)	1.20 (0.97–1.49)	1.27 (1.16–1.39)
Alcoholic Intake	0 ml/wk (*n* = 7,091)	1.00	1.00	1.00	1.00	1.00
	0.23–24.85 ml/wk (*n* = 5,642)	1.22 (1.14–1.32)	1.16 (1.07–1.25)	1.19 (1.09–1.30)	1.18 (0.96–1.47)	1.20 (1.10–1.30)
	24.86–71.69 ml/wk (*n* = 5,654)	1.58 (1.46–1.70)	1.46 (1.35–1.58)	1.48 (1.36–1.61)	1.35 (1.10–1.66)	1.50 (1.38–1.63)
	≥71.70 ml/wk (*n* = 5,623)	1.75 (1.62–1.89)	1.67 (1.54–1.81)	1.65 (1.52–1.80)	1.63 (1.33–2.00)	1.66 (1.52–1.81)
Rank	Junior Enlisted (*n* = 2.231)	1.00	1.00	1.00	1.00	1.00
	Mid–Enlisted (*n* = 10,411)	0.94 (0.84–1.04)	0.94 (0.84–1.05)	0.92 (0.81–1.04)	1.06 (0.76–1.48)	0.93 (0.82–1.05)
	Senior Enlisted (*n* = 3.903)	0.97 (0.84–1.11)	0.91 (0.79–1.05)	1.04 (0.89–1.21)	1.44 (0.96–2.14)	1.06 (0.90–1.23)
	Warrant Officer (*n* = 524)	1.00 (0.80–1.25)	0.81 (0.64–1.02)	1.15 (0.90–1.47)	1.83 (1.06–3.15)	1.18 (0.93–1.51)
	Junior Officer (*n* = 3,628)	1.16 (1.00–1.34)	0.87 (0.75–1.02)	1.44 (1.23–1.69)	1.41 (0.94–2.13)	1.45 (1.24–1.70)
	Senior Officer (*n* = 3,313)	1.18 (1.01–1.38)	0.78 (0.66–0.92)	1.59 (1.33–1.89)	1.65 (1.06–2.58)	1.64 (1.38–1.96)
Occupational Assignment Group	Combat Arms (*n* = 5,990)	1.00	1.00	1.00	1.00	1.00
	Combat Support (*n* = 9,653)	0.84 (0.78–0.90)	0.84 (0.78–0.91)	0.85 (0.78–0.91)	0.79 (0.67–0.94)	0.84 (0.78–0.91)
	Combat Service Support (*n* = 8,367)	0.85 (0.79–0.91)	0.89 (0.82–0.95)	0.78 (0.72–0.85)	0.66 (0.55–0.79)	0.78 (0.72–0.84)
Service	Air Force (*n* = 4,165)	1.00	1.00	1.00	1.00	1.00
	Army (*n* = 7,216)	1.05 (0.98–1.13)	1.17 (1.09–1.26)	0.93 (0.86–1.01)	1.02 (0.84–1.23)	0.93 (0.86–1.01)
	Marine Corps (*n* = 2,889)	1.37 (1.25–1.50)	1.41 (1.29–1.55)	1.38 (1.25–1.53)	1.46 (1.16–1.84)	1.37 (1.25–1.52)
	Navy (*n* = 5,083)	1.15 (1.07–1.24)	1.09 (1.00–1.18)	1.29 (1.18–1.40)	1.28 (1.05–1.57)	1.28 (1.18–1.38)

*Abbreviations*: *SRNS* Sports-related nutritional supplement, *SE* Standard error, *HS* High School, *Grad* Graduate, *Sep* Separated

^a^Service member used either sports bar or sports gel. Included because many military studies combine these supplement types [4–6, 12]

### Adverse effects

Table 4 shows the prevalence of AEs reported by SMs. The overall proportion of SRNS users reporting ≥ 1 AEs was 2.0 ± 0.1 %. In descending order, AEs were most often reported for sport gels, sport bars, and sport drinks. Of those reporting use of one SRNS, 1.8 % reported ≥ 1 AE; of those reporting use of 2 SRNSs, 1.9 % reported ≥ 1 AE; of those reporting use of all 3 SRNSs, 3.8 % reported ≥ 1 AE (p < 0.01).

**Table 4:** Prevalence of adverse effects reported by users of sport-related nutritional supplements

	Adverse Effect	Sport Drinks (*n* = 8,607)	Sport Bars (*n* = 7,167)	Sport Gels (*n* = 895)	Sport Bar &Gel^a^ (*n* = 7,342)
Participants Reporting Specific AEs, %±SE (n)	Palpitations	0.1 ± 0.0 (7)	0.1 ± 0.0 (7)	0.7 ± 0.3 (6)	0.1 ± 0.0 (11)
	Abdominal Pain	0.1 ± 0.0 (12)	0.4 ± 0.1 (28)	0.8 ± 0.3 (7)	0.5 ± 0.1 (35)
	Nausea, Vomiting	0.1 ± 0.0 (9)	0.1 ± 0.0 (8)	0.1 ± 0.1 (1)	0.1 ± 0.0 (9)
	Diarrhea	0.2 ± 0.0 (21)	0.5 ± 0.1 (36)	0.4 ± 0.2 (4)	0.5 ± 0.1 (39)
	Muscle Cramps, Pain, or Weakness	0.1 ± 0.0 (6)	0.1 ± 0.0 (6)	0.2 ± 0.1 (2)	0.1 ± 0.0 (6)
	Sleep Problems, Insomnia	0.2 ± 0.0 (17)	0.1 ± 0.0 (6)	0.4 ± 0.2 (4)	0.1 ± 0.0 (9)
	Dizzy, Confused, Lightheaded	0.1 ± 0.0 (6)	0.0 ± 0.0 (1)	0.3 ± 0.2 (3)	0.0 ± 0.0 (3)
	Tingling, Numbness	0.1 ± 0.0 (9)	0.0 ± 0.0 (1)	0.3 ± 0.2 (3)	0.1 ± 0.0 (4)
	Seizure, Convulsion, Tremor	0.0 ± 0.0 (3)	0.0 ± 0.0 (1)	0.0 ± 0.0 (0)	0.0 ± 0.0 (1)
	Other	0.5 ± 0.1 (45)	0.6 ± 0.1 (41)	0.8 ± 0.3 (7)	0.7 ± 0.1 (48)
Participants Reporting ≥ 1 AE, %±SE (n)		1.3 ± 0.1 (109)	1.6 ± 0.2 (112)	2.8 ± 0.6 (25)	1.8 ± 0.2 (133)

*Abbreviations*: *SE* Standard error, *AE* Adverse effect

^a^Service member used either sports bar or sports gel. Included because many military studies combine these supplement types [4–6, 12]

## Discussion 

This study involving a very large stratified random sample of military SMs found that 45 % of SMs used ≥ 1 SRNS at least once per week and use of sport drinks, bars, and gels was reported by 32, 27, and 3 % of SMs, respectively. Multivariable logistic regression indicated that greater use of any SRNS was independently associated with male gender, younger age, single marital status, more weekly aerobic or resistance training, tobacco use, higher alcohol intake, officer status, combat arms occupations, and service in the Marine Corps or Navy (compared to the Air Force). Overall, the proportion of users reporting ≥ 1 AE was 2.0 %, with 1.3 % for sport drinks, 1.6 % for sport bars, and 2.8 % for sport gels.

### Prevalence

The use prevalence of sport drinks was previously reported in several military and civilian studies. Among Australian soldiers, 42 % reported consuming sport drinks ≥ 1 time/wk [18], and among British soldiers 49 %, reported using sport drinks “currently” [19]. Among young adults in the US (18 to 31 year of age), 31 % reported consuming sport drinks ≥ 1 time/week [20], and in a representative sample of the entire US population (18 to > 65 year of age), 15 % reported sport drink consumption “on a regular and consistent basis” [11]. Previous US military studies [4–6] found sport drinks were used by 24, 23, 51, and 36 % of Air Force, Army, Marine Corps, and Navy personnel, respectively. In these same four services, the current study found use prevalence of 29, 34, 43 and 30 %, respectively. Overall, these data indicate that the prevalence of sport drink use among US SMs is lower than among Australian and British soldiers, similar to young adults in the US, and higher than in the general US population. In making comparisons among prior US military studies [4–6] it should be noted that the Army, Air Force, and Navy/Marine Corps data was collected in 2006–2007, 2010–2011, and 2011–2012, respectively. Thus, these data also suggest that sport drink use may have increased over time in the Air Force and Army while it has decreased in the Marine Corps and Navy.

The prevalence of sport bar and gel use have also been reported previously in several studies. Among Australian soldiers, 6 and 3 % reported use of either sport bars or gels ≥ 1 time/week, respectively [18], and among British soldiers “current” use of these supplements was reported by 18 and 10 %, respectively [19]. About 11 % of the general US population reported use of sport bars on a “regular and consistent basis” in 2011 [11]. Previous studies of US military personnel [4–6] conducted between 2006 and 2012 have combined sport bar and sport gel use and report that 9, 6, 22 and 23 % of Air Force, Army, Marine Corps and Navy personnel, respectively, use these supplements. This compares to 25, 27, 35, and 28 % of these four services in the current study. Overall, these data suggest that sport bar and sport gel use is considerably higher among US soldiers compared to Australian soldiers and the general US population. British soldiers have a lower use of sport bars than US soldiers but higher use of sport gels. Considering the temporal factors mentioned above (i.e., how long ago studies were conducted), these data also suggest use of these supplements has increased over time in all services, especially the Air Force and Army.

A meta-analysis [3] of 18 studies encompassing a wide range of athletic samples found that 44 % (95 %CI = 24–66 %) of male athletes and 35 % (95 %CI = 22–51 %) of female athletes used sport drinks; however, as indicated by the 95 %CIs, there was a very wide range of prevalence depending on the sport and country of origin. The current study found that 34 and 23 % of male and female SMs, respectively, used sport drinks. Summary prevalence estimates from 10 studies reporting on athletes’ use of sport bars found that 28 % (95 %CI = 14–56 %) of male athletes and 32 % (95 %CI = 22–51 %) of female athletes used this supplement [3], compared to 28 and 21 % of men and women in the present study. Thus, compared to a wide range of athletic populations, SM use of sport drinks is, on average, lower. Prevalence of sport bar use among male athletes and male SMs is similar, but female athletes tend to use sport bars more often than female SMs.

### Factors associated with SRNS use

In agreement with the current study, most [5, 6, 11, 12, 18, 21, 22], but not all [4, 11, 22] investigations have been relatively uniform in demonstrating that the proportion of individuals who used sport drinks decreased with age; was higher among men; those who were single; those employed in combat arms occupations; and had little association with BMI. No previous investigation has reported these factors in multivariable analysis, but in the current investigation, these relationships were generally maintained in the logistic regression when all variables were considered together. On the other hand, previous studies [4–6, 11, 12] have not been consistent in finding associations between sport gel/bar use and these factors. In the current study, the relationship between sport bar/gel use was similar to that of sport drinks in that use was highest among younger age groups and was higher among men and those in combat arms occupations. However, in contrast with sport drinks, married SMs and those with BMI in the 25.0-29.9 kg/m^2^ range had the highest use of sport bars/gels. Marine Corps personnel had the highest use of all three SRNS types in both univariate and multivariable analyses.

Previous studies [4–6, 18, 21, 22] have been generally consistent in reporting that sport drink and sport bar/gel use increases as physical activity increases, in agreement with the current study. Military personnel are much more physically active compared to civilian populations [23, 24] because they must acquire and maintain a relatively high level of physical fitness to pass regular physical fitness tests [25] and because of heavy physical requirements in many occupational specialties [26]. The fact that use of all types of SRNSs was higher as the duration of training increased is likely because SRNSs are often used in connection with sport and exercise activity and as individuals train more, they are more likely to use SRNSs. Interestingly, in the current study sport gel use was substantially higher with higher levels of aerobic or resistance training in the univariate analysis. However, in multivariable analysis the relationship between sport gel use and resistance training duration was severely attenuated while that with aerobic exercise was considerably enhanced. This suggested that aerobic exercise duration had a much stronger relationship with sport gel use compared to resistance training duration.

In the current study, tobacco users (current smoker or smokeless tobacco user) were more likely to use SRNSs, and as alcohol consumption increased there was increased use of all three types of SRNSs. In multivariable analysis, the relationship between smoking and sport bar and sport gel use was no longer significant, but the other relationships were maintained. A number of studies that have examined smoking and SRNSs have shown little association between smoking and sport drink or sport bar/gel use [4, 12, 20], but two investigations [21, 22] found adolescent smokers were about twice as likely as nonsmokers to use sport drinks. The reasons for the conflicting results are not apparent. In univariate and multivariable analyses in the current study, alcohol use demonstrated a strong and dose-related relationship with sport drinks, bars, and gels in that use of these SRNSs was higher with increasing levels of alcohol consumption. No previous studies could be found on this relationship, suggesting further research is required.

### Adverse effects

In the present study, the prevalence of self-reported AEs for all types of SRNSs was low with 1.3, 1.6, and 2.8 % of SMs reporting ≥ 1 AE for sport drinks, bars, and gels, respectively. In a previous study of Navy and Marine Corps personnel, 3.7 % of participants reported ≥ 1 AE for sport drinks and 3.1 % for sports bars/gels [6]. The AE prevalence for SRNSs is in contrast to dietary supplements for which 8–29 % of military SMs have previously reported AEs, but largely using convenience samples [6, 13, 27–33]. Data recently collected on a large stratified random sample of SMs reported that 18 % of DS users reported AEs, near the middle of this range [13]. Nutritional supplements like SRNSs are regulated by the FDA as foods and are subject to the regulatory framework of the Federal Food, Drug, and Cosmetic Act of 1938, and the numerous modifications to that act since 1938 [34, 35]. This legislation prescribes that no substance can be introduced into the US food supply unless it has been “generally recognized as safe” meaning that “there is a reasonable certainty in the mind of competent scientists that the substance is not harmful under its intended conditions of use” [36]. By statute, a “Nutrition Fact” label is required on the food packaging, and that label must contain all ingredients by their common name and in descending order of amount [36]. In contrast, DSs are controlled under the DSHEA of 1994 [8], which gives the FDA limited regulatory authority. Manufacturers must notify the FDA before marketing a new DS, but FDA approval is not required for retailing the product. The FDA has the burden of demonstrating a specific product is unsafe, although manufacturers are required to notify the FDA about serious AEs [37, 38]. A “Supplement Facts” label is required on DS packaging that must show the ingredients in the supplement [39], but for proprietary blends, amounts of the ingredients are not required. The low incidence of AEs among SRNSs examined here suggest regulation as “foods” results in a lower incidence of AEs compared with DSs which are regulated separately.

### Strengths and limitations

The current study had the advantage of employing a very large random sample of SMs that was stratified on known demographics of gender and military service. The questionnaire was standardized and based on previous questionnaires designed for obtaining similar supplement data from SMs [16]. Nonetheless there were some limitations to this study. All data were self-reported and suffer the usual weaknesses associated with this method including recall bias, social desirability, errors in self-observation, and inadequate recall [40, 41]. An attempt was made to mitigate some of these factors by requesting SRNS use only in the last 6 months. In self-reporting AEs, participants were limited to nine AE categories, although they could report and describe other AEs that were subsequently categorized by the investigators. Matching SRNS use with AEs reported in clinical medical records would have provided a more definitive description of the AEs.

## Conclusions

The current investigation of a large (*n* = 26,681), stratified random sample of military personnel found sport drinks, sport bars, and sport gels were used by 32, 27, and 3 % of SMs at least once per week, respectively. Multivariable logistic regression indicated greater use of any SRNS was independently associated with male gender, younger age, single marital status, more weekly aerobic or resistance training, tobacco use, higher alcohol intake, officer status, and service in the Marine Corps or Navy (compared to the Air Force). The proportion of users reporting ≥ 1 AE was 1.3 % for sport drinks, 1.6 % for sport bars, and 2.8 % for sport gels. Use of SRNSs among SMs was high and self-reported AEs comparatively low. The more stringent regulation of these sport products may account, at least in part, for their greater apparent safety, at least with regard to the self-report of AEs. These data emphasize the high prevalence of SRNS use among SMs [4–6] and the relatively low incidence of self-reported AEs particularly in comparison to many categories of DSs. The latter suggests that SRNSs are safe to use.

## Data Availability

The datasets generated and/or analyzed during the current study are not publicly available due to US government restrictions, but are available from the corresponding author on reasonable request.

## Abbreviations

95%CI: 95% confidence interval
AE: Adverse effect
BMI: Body mass index
DMDC: Defense Manpower Data Center
DS: Dietary supplement
DSHEA: Dietary Supplement and Health Education Act
FDA: Food and Drug Administration
OR: Odds ratio
SE: Standard error
SM: Service member
SRNS: Sports-related nutritional supplement
US: United States
